# Estimation of the size of the Ebola outbreak caused by Bundibugyo virus in the Democratic Republic of the Congo

**DOI:** 10.1016/S1473-3099(26)00299-9

**Published:** 2026-06-09

**Authors:** Ruth McCabe, Lennox Ebbarnezh, Solome Okware, Richard Fotsing, Etien Koua, Paul Mbaka, Adelard Lofungola, Dav M Ebengo, Placide K Mbala, Tania T Bishola, Christian M. Ibolobolo, Herman M. Matondo, Jean-Clement M Sibo, Sabine L van Elsland, Martina McMenamin, Neil M Ferguson, Olivier le Polain de Waroux, Anne Cori

**Affiliations:** 1 MRC Centre for Global Infectious Disease Analysis, WHO Collaborating Centre for Infectious Disease Modelling, and Jameel Institute, School of Public Health, Imperial College London, UK; 2 WHO Health Emergencies Programme, Geneva, Switzerland; 3 World Health Organization Country Office for Uganda Kampala, Uganda; 4 World Health Organization Country Office for the Democratic Republic of the Congo, Kinshasa, Democratic Republic of the Congo; 5 World Health Organization Regional Office for Africa, Brazzaville, Congo; 6 Uganda Ministry of Health, Kampala, Uganda; 7 Institut National de Santé Publique (INSP), Kinshasa, Democratic Republic of the Congo; 8 One Health Institute for Africa (INOHA), University of Kinshasa, Kinshasa, Democratic Republic of the Congo; 9 Modeling and Epidemic Intelligence Unit, Department of Epidemiology and Global Health, Institut National de Recherche Biomédicale, Kinshasa, Democratic Republic of the Congo; 10 Department of Medical Biology, Microbiology Service, University Clinics of Kinshasa, University of Kinshasa, Kinshasa, Democratic Republic of the Congo; 11 Department of Life Sciences, Faculty of Sciences and Technology, University of Kinshasa, Kinshasa, Democratic Republic of the Congo

The ongoing outbreak of Bundibugyo virus disease (BVD), a form of Ebola virus disease caused by the Bundibugyo virus, in the Democratic Republic of the Congo (DRC) is evolving rapidly. As of 27 May 2026, a total of 1,031 suspected or confirmed cases of BVD had been reported from fourteen health zones across three provinces (Ituri, Nord Kivu, Sud Kivu) DRC including 240 suspected or confirmed deaths.^1^ Testing has rapidly expanded for routine and retrospective assessment of suspected cases (381 confirmed cases as of 3 June 2026). Deaths are harder to retrospectively assess, with investigations ongoing (64 confirmed deaths as of 3 June 2026). Suspected cases and deaths are no longer reported in recent situation reports.^2^ An additional sixteen cases were confirmed in Uganda, as of 4 June 2026,^3^ with three of those among individuals travelling from Ituri Province, DRC (Uganda Ministry of Health 23 May 2026; World Health Organization 29 May 2026).^4,5^ Together, these observations suggest that the epidemic is larger than currently ascertained; however, its true magnitude remains uncertain. Estimating the outbreak size is important to assess the scale of the public health threat and to appropriately calibrate surveillance and response efforts.

To produce early estimates of the current size of the epidemic in the DRC as of 27 May 2026, we conducted scenario-based analyses using two simple, independent approaches, leveraging the limited information currently available and making plausible assumptions about the surveillance, severity, and growth of the outbreak (full methodological detail in Appendix).

The first method uses an analytical framework linking reported suspected and confirmed deaths, of which we assume a proportion is due to Ebola, with estimates of the case fatality ratio (CFR; proportion of cases who die as a result of a Bundibugyo infection; 33% 95% CI 26-40%) and the time from symptom onset to death (gamma distributed, mean 11.37 days, standard deviation 5.41 days) derived from previous Bundibugyo virus outbreaks.^6,7^ We further assume exponential epidemic growth following a single zoonotic spillover event.^8^ Assuming 30% of suspected and confirmed deaths are due to Ebola virus disease, a case fatality ratio of 33% and a doubling time of 10 days yields a mean estimated outbreak size of 451 cases (95% CI 396–511) as of 27 May 2026 (Table 1). Estimated outbreak size increases with assumptions of a higher proportion of deaths due to Ebola, a lower case fatality ratio, and fast epidemic growth.

The second method extends the method described by Imai et al.^9^. We combine information on the three confirmed imported BVD cases reported in Uganda (as of 27 May 2026); estimates of daily international travel volumes from Ituri and Nord Kivu provinces to Uganda; assumptions about the epidemic doubling time, and we presumed that all cases in symptomatic travellers will be detected at the time of recovery or death.^6,7^ Assuming Ituri province as the source population, and a doubling time of 10 days, this approach yields an estimated outbreak size of 945 (95% CI 196-2,274**)** cases. Expanding the source population to further include Nord Kivu increases these estimates (Table 2). However, these findings should be interpreted with caution, as at least two of the three imported cases reportedly travelled to Uganda specifically to seek medical care.^10^ Assuming fewer travellers reduces the epidemic size accordingly (Appendix).

The two simple methods yield broadly consistent results, suggesting that as of 27 May 2026, approximately 280 to 2,520 cases of BVD may have occurred in the DRC (estimate range: 306 – 2,521 under death back-calculation model; 282 – 1,345 under geographical spread model). However, there is considerable uncertainty around these estimates. Nonetheless, the broad convergence of estimates from two independent methods supports the conclusion of considerable under-detection and the potential for wider transmission.

As with all modelling approaches, these estimates rely on several key assumptions: that transmission is largely concentrated in Ituri and Nord Kivu provinces; the scale and patterns of population movement from these areas to Uganda are accurately captured within the point of entry data used; the percentage of deaths attributable to Ebola is between 30% and 100%; the CFR and time between onset and death in this outbreak are consistent with those derived from past Bundibugyo virus outbreaks; and the current epidemic growth rate is between 7 - 14 days, loosely based on early genomic analyses.^11^ Each of these assumptions is subject to substantial uncertainty which may influence the resulting estimates. A full list of caveats is provided in the appendix. We underline that this is an early analysis, primarily conducted to inform operational planning, and that estimates will change as more data and information about the outbreak become available.

To guide response efforts, it is important that the size, growth rate and geographic extent of the epidemic are better characterised as soon as possible; both epidemiological (e.g. case time series) and genomic data (informing estimates of the time of the most recent common ancestor, e.g. Mbala et al. 2026) will be critical to achieve this goal.

These findings point to potentially substantial undetected transmission of BVD in eastern DRC. Importantly, these estimates highlight the large human burden of the outbreak. Rapid, targeted public health interventions, urgent medical support for affected and at-risk populations and strong international coordination are essential.

## Supplementary Material

Appendix

## Figures and Tables

**Table 1:** Estimated outbreak sizes derived using method 1 (backcalculation from 240 reported suspected and confirmed deaths), under 3 assumptions for the epidemic growth rate (a main scenario with a 10-day doubling time, and two sensitivity analyses with 7- and 14- day doubling times) and 3 estimates of the CFR. 95% confidence intervals derived assuming Poisson variation shown. The results assuming 100% of suspected and confirmed deaths are due to Ebola virus disease are presented in the Appendix.

Scenario	Doubling time (days)	Corresponding growth rate (per day)	Number of cases: Mean (95%CI)		
			CFR = 26%	CFR = 33%	CFR = 40%
**Main scenario:** Moderate growth, intermediate emergence	10	0.07	**573** **(502 - 648)**	**451** **(396 - 511)**	**372** **(327 - 421)**
**Sensitivity analysis 1:** Fast growth, recent emergence	7	0.1	**756** **(664 - 855)**	**596** **(523 - 674)**	**491** **(432 - 556)**
**Sensitivity analysis 2:** Slow growth, older emergence	14	0.05	**471** **(413 - 533)**	**371** **(326 - 420)**	**306** **(269 - 346)**

**Table 2:** Estimated outbreak size derived using method 2 (geographic spread), under two assumptions regarding the source populations (main assumption: source population = Ituri, sensitivity analysis: source population = Ituri + Nord Kivu), assuming all cases in symptomatic travellers will be detected by the time of recovery or death, given three observed imported cases into Uganda. Confidence intervals (CIs) calculated as exact negative binomial CIs. The results assuming only one imported case are shown in the Appendix.

Scenario	Doubling time	Corresponding growth rate	Number of cases: Mean (95% CI)	
			Ituri province Population: 4,392,200	Ituri and Nord Kivu provinces Population: 12,593,600
**Main scenario:** Moderate growth, intermediate emergence	10	0.07	**945 (196 - 2,274)**	**1,164 (241 - 2,800)**
**Sensitivity analysis 1:** Fast growth, recent emergence	7	0.1	**1,100 (228 - 2,647)**	**1,354 (281 - 3,260)**
**Sensitivity analysis 2:** Slow growth, older emergence	14	0.05	**847 (176 - 2,037)**	**1,042 (216 - 2,508)**

## Data Availability

All code to reproduce these analyses can be found at: https://github.com/mrc-ide/evd-2026-outbreak-size.
