# Supplementary material for: Estimation of the size of the Ebola outbreak caused by Bundibugyo virus in the Democratic Republic of the Congo
Source: Lancet Infect Dis. Author manuscript; Available in PMC 2026 Sep 4. (PMC7619448; doi:10.1016/S1473-3099(26)00299-9)
Supplement: Supplementary file 1 [file EMS218038-supplement-Appendix.pdf]

Appendix

Estimation of the size of the Ebola outbreak caused by Bundibugyo virus in the Democratic Republic of the Congo

Ruth McCabe[1], Lennox Ebbarnezh[2], Solome Okware[3], Richard Fotsing[4], Etien Koua[5], Paul Mbaka[6], Adelard Lofungola[7], Dav M Ebengo[8,9], Placide K Mbala[9,10], Tania T Bishola[8,9,11], Christian M Ibolobolo[9], Herman M Matondo[8,9], Jean-Clement M Sibo[8,9], Sabine L van Elsland[1], Martina McMenamin[2], Neil M Ferguson[1], Olivier le Polain de Waroux[2,*], Anne Cori[1,*,+]

[1]MRC Centre for Global Infectious Disease Analysis and Jameel Institute, School of Public Health, Imperial College London, London, UK

[2]World Health Organization Health Emergencies Programme, Geneva, Switzerland

[3]World Health Organization Country Office for Uganda

[4]World Health Organization Country Office for the Democratic Republic of the Congo

[5]World Health Organization Regional Office for Africa

[6]Uganda Ministry of Health

[7]Insitut National de Santé Publique (INSP), Democratic Republic of the Congo

[8]One Health Institute for Africa (INOHA), University of Kinshasa, Democratic Republic of the Congo

[9]Modeling and Epidemic Intelligence Unit, Department of Epidemiology and Global Health, Institut National de Recherche Biomédicale, Kinshasa, Democratic Republic of the Congo

[10]Department of Medical Biology, Microbiology Service, University Clinics of Kinshasa, University of Kinshasa, Kinshasa, Democratic Republic of the Congo

[11]Department of Biology, Faculty of Science and Technology, University of Kinshasa, Democratic Republic of the Congo

[*]Joint senior authors

[+] Corresponding author: a.cori@imperial.ac.uk

Methods

We used two independent analyses to estimate the outbreak size; the first relies on the total number of suspected BVD deaths reported and estimates of the case fatality ratio (CFR) and time between symptom onset and death; the second relies on the number of confirmed cases reported outside the Democratic Republic of the Congo and population movement information. Both methods share some common simplifying assumptions:

- the outbreak was seeded T days ago with a single zoonotic case in the human population;
- the outbreak has been growing exponentially since that first case, with doubling time τ_2.

Under these assumptions, the cumulative incidence of cases by time T after the outbreak start is

$$C_T = e^{rT},$$

where $r = \ln(2)/\tau_2$ is the epidemic growth rate. The daily incidence also grows exponentially at rate r:

$$I_T = \frac{dC_T}{dt} = re^{rT}.$$

Method 1: Backcalculation from deaths

The first analysis backcalculates the number of BVD cases from the total number of suspected and confirmed BVD deaths reported (240 as of 27 May 2026 [1]), using previously published estimates of the distribution of time from symptom onset to death in BVD cases [2]. It examines the sensitivity of estimates to:

- the assumed doubling time and start date of the epidemic
- the case fatality ratio (CFR, proportion of cases who die as a result of their infection) for Bundibugyo virus.

We estimate the total epidemic size using an analytical formulation that links the cumulative number of cases (counted by date of symptom onset) to the cumulative number of deaths under the assumption that the delay from symptom onset to death is gamma distributed with mean 11.37 days and standard deviation 5.41 days, as estimated by Rosello et al. [2] in the 2012 Bundibugyo outbreak in Isiro, Democratic Republic of the Congo. These values imply shape and rate parameters $\alpha = 4.42$ and $\beta = 0.388$/day.
If $f(u)$ is the probability density function of the delay from symptom onset to death, then the expected cumulative number of deaths by time T is

$$D_T = \theta \int_0^T \int_0^{T-t} I_t f(s)\, ds\, dt,$$

where θ is the CFR. Substituting $I_t = re^{rt}$, reordering the integrals and integrating the exponential gives:

$$D_T = \theta \int_0^T e^{rt} f(T-t)\, dt.$$

Assuming a gamma distribution for $f(u)$ with shape α and rate β, this evaluates to

$$D_T = \theta\, C_T (1 + r/\beta)^{-\alpha}\, \frac{\gamma(\alpha, (\beta + r)T)}{\Gamma(\alpha)},$$

where Γ is the gamma function and γ is the lower incomplete gamma function. For the parameter values and scenarios explored here, this expression is well approximated by

$$D_T \approx \theta\, C_T (1 + r/\beta)^{-\alpha}.$$

Rearranging gives an estimator for the cumulative number of cases:

$$C_T = D_T (1 + r/\beta)^{\alpha}/\theta.$$

The scaling by the reciprocal of the CFR is to be expected: for the same number of cumulative deaths, one would expect proportionally fewer cases as the CFR increases. The term $(1 + r/\beta)^\alpha$ represents the effect of epidemic phase bias—that in a growing epidemic, a disproportionate number of cases will have been infected recently, and therefore will not yet have died (or recovered).

We examine three values of the CFR, of 26.4%, 33.0% and 40.0%, spanning the central estimate and 95% confidence bands of published estimates of the two previous BVD outbreaks according to the United States Centers for Disease Control and Prevention (55 deaths, 169 cases) [3].

We note that the assumed duration of the outbreak, T, constrains what doubling time (or equivalently, epidemic growth rate, r) is consistent with the observed epidemic, since $C_T = e^{rT}$, meaning $T = \ln(C_T)/r$. Hence choosing a particular doubling time (and thus growth rate) determines both the total number of cases C_T and the duration of the epidemic T, under our assumption of exponential growth from a single initial case. Here we consider three values of the doubling time: 7, 10 and 14 days.

In deriving confidence intervals for Table 1, we conservatively assume D_T is Poisson distributed with mean $C_T(1 + r/\beta)^{-\alpha}$ and calculate the likelihood profile for C_T for the three assumed values of r.

Method 2: Geographic Spread

We use a refined version of the analysis given in Imai et al. [4]. We estimate the number of BVD cases in the Democratic Republic of the Congo using information on:

- the reported number of confirmed BVD cases in Uganda (three as of 22 May 2026);
- the estimated daily number of international travellers from the Ituri and Nord Kivu Provinces in the Democratic Republic of the Congo to Uganda;
- assumptions about how long after infection individuals are detected.

If X_T is the number of cases detected in Uganda by time T after the start of the outbreak, and $G(s)$ is the probability of infection in a traveller being detected time s after symptom onset, then following similar logic as used for Method 1, it can be shown that

$$X_T = \rho \int_0^T e^{r(T-s)} G(s)\, ds,$$

where ρ is the daily probability of travel to Uganda, given by

$$\rho = \frac{\text{daily outbound international travellers from source population}}{\text{source population size}}.$$

Assuming (conservatively) that all symptomatic cases are detected if they travel before death or recovery, $G(s) = 1 - H(s)$ where $H(s)$ is the cumulative distribution function for the disease onset to disease outcome distribution. We model $H(s)$ as a gamma distribution with shape and rate parameters α and β respectively, as in method 1, thereby assuming that the onset to death and onset to recovery delay have similar distribution, in line with estimates from Wamala et al. [5]. It follows that:

$$X_T \approx \frac{\rho}{r} C_T \left(1 - (1 + r/\beta)^{-\alpha}\right),$$

where the approximation is excellent if T is much larger than the mean time from onset to outcome (i.e. $T \gg \alpha/\beta$). Rewriting this gives:

$$C_T \approx \frac{r X_T}{\rho\,(1 - (1 + r/\beta)^{-\alpha})}.$$

To calculate confidence intervals, we model the number of cases which were not exported, $C_t - X_T$ as a negative binomial distribution with the number of successes parameter set to X_T and the probability of success, p_1, given by:

$$p_1 \approx \frac{\rho\,(1 - (1 + r/\beta)^{-\alpha})}{r}.$$

As a sensitivity analysis, we also examine the scenario where infection in travellers is always detected from the time of *infection* (rather than just disease onset) to when disease is resolved. Given our

analysis focuses on modelling symptomatic cases, this requires some adjustment of the formalism above. Assuming the infection to outcome distribution can also be well described by a gamma distribution with shape and rate parameters α_2 and β_2 respectively, and that the incubation period distribution can be similarly described by a gamma distribution with parameters s α_3 and β_3, it can be shown that

$$C_T \approx \frac{rX_T(1+r/\beta_3)^{-\alpha_3}}{\rho\,(1-(1+r/\beta_2)^{-\alpha_2})}.$$

Here we use $\alpha_2 = 6.93$ and $\beta_2 = 0.39$, corresponding to a mean infection to outcome delay of 17.7 days and a variance of 45.1 days^2. These were obtained as the respective sums of the mean and variances of the incubation period (mean 6.3, variance 15.8 (giving $\alpha_3 = 2.52$, $\beta_3 = 0.40$) derived from the reported confidence intervals in [6]) and of the onset to outcome delay (mean 11.4, variance 29.3 [2]).
The term $(1+r/\beta_3)^{-\alpha_3}$ represents the proportion of cumulative infections which are or have previously been symptomatic, and is necessary to translate (in a growing epidemic) from infections (some of whom will not yet have developed symptoms) to symptomatic cases. Then confidence intervals are calculated by compounding a binomial distribution with sampling probability $(1 + r/\beta_3)^{-\alpha_3}$ with a negative binomial distribution for the total number of infections, giving another negative binomial distribution with modified success probability. Hence the number of cases which were not exported, $C_T - X_T$ is negatively binomially distributed with the number of successes parameter again set to X_T but a modified probability of success, p_2, given by:

$$p_2 \approx \frac{\rho\,(1-(1+r/\beta_2)^{-\alpha_2})}{r(1+r/\beta_3)^{-\alpha_3}}.$$

We take $X_T = 3$, and consider a total of four scenarios, assuming:

- one of two travel probabilities derived from two source population sets [7]:
  1. Ituri only (population 4,392,000),
  2. Ituri + Nord Kivu (population 13,392,200);
- the two assumptions about detection discussed above:
  1. all infections in symptomatic travellers will be detected, until symptoms resolve;
  2. all infections in both asymptomatic (incubating) and symptomatic travellers will be detected, until symptoms resolve (not shown in main text).

For each of these, we examine the three doubling times examined for Method 1: 7, 10 and 14 days.
Table S1 presents the sensitivity analysis for the geographic spread model, assuming that all cases in affected travellers (incubating or symptomatic), will be detected at the time of travel (a deliberately extreme scenario).

Probability of travel

The probability of travel is estimated from point-of-entry (PoE) data shared by WHO. Traveller screening data were available for the following PoEs along the Uganda–Democratic Republic of the Congo border: Busanza, Busunga, Goli, Mpondwe, Ntoroko Main, Odramacaku, and Vurra.
We received data from four situation reports (sitreps), each covering a one-week period. Some PoEs were not covered in all sitreps. The data correspond to:

- epiweek 10 (2–8 March 2026),
- epiweek 11 (9–15 March 2026),
- epiweek 15 (6–12 April 2026),
- epiweek 18 (27 April–3 May 2026).

We further split PoEs by whether the corresponding province on the DRC side was Ituri or Nord Kivu. The data used in our analyses are reproduced in Table S2.
Population size estimates for each province were taken from estimates by City Population [7].

Table S1: Estimated outbreak size estimated by method 2 (geographic spread), under the 2 source populations (main assumption: source population = Ituri, sensitivity analysis: source population = Ituri + Nord Kivu), under the extreme assumption all cases in affected travellers (incubating or symptomatic), will be detected at the time of travel, given three observed imported cases into Uganda. Confidence intervals (CIs) were calculated as exact negative binomial CIs.

**Scenario**	**Doubling time (days)**	**Growth rate**	**Number of cases (mean (95% CIs))**	
			**Ituri province Population: 4,392,000**	**Ituri + Nord Kivu Population: 12,539,600**
Main scenario: Moderate growth intermediate emergence	10	0.07	350 (73–841)	431 (90–1,036)
Sensitivity analysis 1: Fast growth recent emergence	7	0.10	474 (99–1,139)	583 (121–1,403)
Sensitivity analysis 2: Slow growth older emergence	14	0.05	278 (59–688)	343 (72–823)

Table S2: Summary of passenger data underpinning estimates.

**Location**	**Point of entry (PoE)**	**Number of sitreps with an observation**	**Mean reported weekly passengers entering Uganda through this PoE**	**Mean daily passengers***	**Total mean daily passengers****
Ituri	Goli	4	4,150	593	1,871
	Ntoroko Main	4	1,846	264	
	Odramacaku	2	3,137	448	
	Vurra	4	3,964	566	
Nord Kivu	Busanza	1	919	131	2,468
	Busunga	3	9,158	1,308	
	Mpondwe	4	7,326	1,047	

* Mean daily passengers derived by dividing weekly totals by 7 and averaging across sitreps.
** Total mean daily passengers rounded to the nearest integer at the final analysis step.

Caveats

- Method 1 uses a simple deterministic epidemic model to explore the relationship epidemic growth rate and epidemic size to date; it does not fully capture stochasticity or overdispersion (superspreading) in the underlying processes and therefore underestimates true uncertainty.
- There is high uncertainty in method 2 estimates as these are based on only three exported cases, at least two of which travelled specifically for the purposes of seeking care.
- Estimates of the CFR, incubation period, and time from symptom onset to death or recovery for BVD are scarce [8] - we are using the limited available evidence from previous outbreaks and did not account for uncertainty in the estimated distributions of epidemiological delays.
- In particular, Method 2 relies on characterising the case detection window which is challenging for Bundibugyo: the mean incubation period has been estimated to be 6–7 days [5, 6] but the onset to detection is not well characterised for this species [8].
- The data on border crossing is not comprehensive and may miss informal crossings. If so, the epidemic size might be lower than the estimates presented here. Moreover, the PoE numbers we are using covers other social service seeking including educational, family, religious. It also includes border community movements which may introduce bias.

- There is some indication that at least two of the three cases detected in Uganda had travelled there for treatment. This might indicate that cases have a higher probability of travel than the general population, which would bias our Method 2 estimates of epidemic size upwards.
- Method 1 relies on the reported number of suspected BVD deaths detected through syndromic surveillance, which is imperfect. These figures could both be an over- or under-estimate of deaths actually attributable to BVD.
- In the absence of data on the timing of cases or deaths, the growth rate and date of origin of the current epidemic cannot be estimated. In both methods, we therefore explore a range of plausible assumptions for these parameters, loosely based on recently published estimates of the time of the most recent ancestor based on the first published BVD genomes from this epidemic [9]. Assumptions around the epidemic growth rate could be refined as more detailed data become available, either to better characterise the likely start of the epidemic with additional genomic data, or to estimate growth rates from time series of clinical outcomes.
- Both methods consider constant rather than changing parameter inputs, such as the CFR and doubling time, and do not explicitly account for other important variables driving this epidemic (e.g., case follow-up, increased surveillance).
- Here we have estimated the outbreak size in terms of symptomatic cases throughout, given that available data report symptomatic cases (rather than providing information on pre-symptomatic infections). It should be noted that in an exponentially growing epidemic, the total number of infections (including infected individuals in their incubation period) would be expected to be substantially larger (by a factor $(1 + r/\beta_3)^{\alpha_3}$, where α_3 and β_3 are the parameters of a gamma-distributed incubation period distribution - see above).

Code availability

All code to reproduce these analyses can be found at: `https://github.com/mrc-ide/evd-2026-outbreak-size`.

References

[1] Institut National de Sante Publique. *SitRep MVE N° 013/2026*. Available from: `https://insp.cd/sitrep-mve-n-013-2026/`. 2026.

[2] A Rosello et al. "Ebola virus disease in the Democratic Republic of the Congo, 1976-2014". en. In: *Elife* 4 (2015), e09015.

[3] US Center for Disease Control. *Ebola Disease Outbreaks by Species and Size, Since 1976*. Available from: `https://www.cdc.gov/ebola/outbreaks/index.html`.

[4] N Imai et al. *Report 1: Estimating the potential total number of novel Coronavirus cases in Wuhan City, China*. 2020.

[5] JF Wamala et al. "Ebola hemorrhagic fever associated with novel virus strain, Uganda, 2007-2008". en. In: *Emerg. Infect. Dis.* 16.7 (2010), pp. 1087–1092.

[6] A MacNeil et al. "Proportion of deaths and clinical features in Bundibugyo Ebola virus infection, Uganda". In: *Emerg. Infect. Dis.* 16.12 (2010), pp. 1969–1972.

[7] *Democratic Republic of the Congo (formerly Zaire)*. City Population. Available from: `https://www.citypopulation.de/en/drcongo/cities/`. 2026.

[8] RK Nash et al. "Ebola virus disease mathematical models and epidemiological parameters: a systematic review". In: *Lancet Infect. Dis.* 24.12 (2024), e762–e773.

[9] P Mbala et al. *Initial genomes from May 2026 Bundibugyo Virus Disease Outbreak in the Democratic Republic of the Congo and Uganda, reveal a new spillover event*. Available from: `https://virological.org/t/initial-genomes-from-may-2026-bundibugyo-virus-disease-outbreak-in-the-democratic-republic-of-the-congo-and-uganda/1032`. 2026.
